# PAK in Pancreatic Cancer-Associated Vasculature: Implications for Therapeutic Response

**DOI:** 10.3390/cells12232692

**Published:** 2023-11-23

**Authors:** Arian Ansardamavandi, Mehrdad Nikfarjam, Hong He

**Affiliations:** 1 Department of Surgery, Austin Precinct, The University of Melbourne, 145 Studley Rd, Heidelberg, VIC 3084, Australia; aansardamava@student.unimelb.edu.au (A.A.); m.nikfarjam@unimelb.edu.au (M.N.); 2 Department of Hepatopancreatic-Biliary Surgery, Austin Health, 145 Studley Rd, Heidelberg, VIC 3084, Australia

**Keywords:** angiogenesis, pancreatic cancer, vessel normalisation, p21-activated kinases (PAKs), immunotherapy, chemotherapy

## Abstract

Angiogenesis has been associated with numbers of solid tumours. Anti-angiogenesis drugs starve tumours of nutrients and oxygen but also make it difficult for a chemo reagent to distribute into a tumour, leading to aggressive tumour growth. Anti-angiogenesis drugs do not appear to improve the overall survival rate of pancreatic cancer. Vessel normalisation is merging as one of the new approaches for halting tumour progression by facilitating the tumour infiltration of immune cells and the delivery of chemo reagents. Targeting p21-activated kinases (PAKs) in cancer has been shown to inhibit cancer cell growth and improve the efficacy of chemotherapy. Inhibition of PAK enhances anti-tumour immunity and stimulates the efficacy of immune checkpoint blockades. Inhibition of PAK also improves Car-T immunotherapy by reprogramming the vascular microenvironment. This review summarizes current research on PAK’s role in tumour vasculature and therapeutical response, with a focus on pancreatic cancer.

## 1. Introduction

The global incidence of pancreatic ductal adenocarcinoma (PDA) is increasing. Pancreatic cancer now ranks among the top ten most prevalent cancers [1]. The poor prognosis of PDA is mainly due to the lack of an early detection method and no effective systemic therapy for metastatic and locally advanced diseases [2]. Less than a third of patients receive surgical resection, of which 65% of patients experience tumour recurrence after surgical resection [3].

One of the main barriers to developing effective treatments for PDA is its unique characteristic of the tumour microenvironment (TME). The TME of PDA exhibits a dense stromal environment, containing cancer-associated fibroblasts and immune cells that interact with different subsets of cancerous cells, including cancer stem cells (CSCs) [4]. In addition, the blood vessels in the TME facilitate the exchange of nutrients, oxygen, and other essential factors and provide pathways for the dissemination of tumour cells to distant sites [5]. Interactions between blood vessels and other elements of the TME influence overall tumour behaviour and therapeutic response [6].

Inhibiting blood vessels may limit the expansion of the tumour and enhance the survival of cancer patients [7,8]. In 2004, FDA approved the clinical application of bevacizumab, an anti-VEGF antibody, to be used as an initial therapeutic approach for metastatic colorectal cancer (5-FU) [9]. Furthermore, FDA has approved multi-target tyrosine kinase inhibitors (TKIs) like sunitinib, sorafenib, and pazopanib. These TKIs are designed to specifically target VEGF receptors, with a particular focus on VEGFR-2 receptor [10]. In pancreatic cancer treatment, various clinical trials have been conducted to assess the efficacy of anti-angiogenic agents. However, the outcomes have been disappointing across multiple studies [11]. Although a few clinical trials have shown improvements in progression-free survival (PFS) [12], none of them have indicated any significant extension in the overall survival (OS) of pancreatic cancer patients. The use of anti-angiogenic treatment for pancreatic cancer remains a subject of debate [13]. Nevertheless, an increasing body of clinical evidence suggests that a substantial number of tumours exhibit an initial lack of response to anti-angiogenic agents, termed de novo resistance, while others gradually acquire resistance over time, resulting in tumour progression even after several months of treatment [14]. Although high microvessel density (MVD) is generally considered to be a predictor of poor prognosis [15,16], several studies found no correlation between poor prognosis and high MVD [17,18]. PDA exhibits limited response to traditional chemotherapy treatments because of the low MVD. Instead of the inhibition of blood vessels, vascular normalisation holds potential promise as an innovative approach for cancer treatment [19], especially for pancreatic cancer.

Over 90% of PDA carry KRas mutations [20]. KRas is a member of the Ras family proteins that bind to guanosine triphosphate (GTP), becoming active. When mutated, KRas becomes constitutively active, triggering the activation of downstream pathways critical for cell survival and growth [21]. Through direct and indirect mechanisms, KRas activates p21-activated kinases (PAKs) [22]. PAKs have gained considerable attention for their roles in the tumorigenesis of PDA [23]. Recent studies have also revealed PAK’s involvement in tumour vascular networks and immune response. In this review, we will explore the potential connections between PAK, vascularisation, and its impact on therapeutical response in pancreatic cancer.

## 2. Vascular Network and Therapeutic Responsiveness

**Figure 1:**
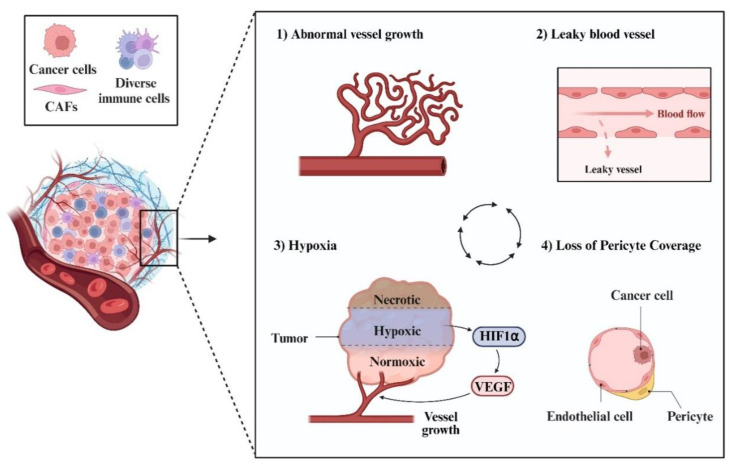
Factors contributing to abnormal tumour vasculature. One of the essential components within the tumour microenvironment (TME) is the existence of abnormal vascular structures. Abnormal blood vessel growth, leaky vessel, loss of pericyte support, and low levels of oxygen are primary characteristics of these blood vessels. These features are interconnected and can mutually enhance the abnormalisation of blood vessels. For instance, a hypoxic microenvironment triggers the production of VEGF, which in turn reinforces the growth of abnormal blood vessels [27].

Tumour blood vessels exhibit many defects, such as abnormal vessel growth, leaky structure with an absence of proper pericyte support, and loss of the normal structures found in arterioles, capillaries, and venules [24]. These defects of tumour blood vessels compromise perfusion, which in turn leads to a hypoxic and acidic microenvironment within the tumour [25]. These factors and/or events interact with each other (Figure 1) [9] and collectively hinder the effective delivery of chemotherapeutic agents [26], contributing to tumour growth, survival, and metastasis.

**Figure 2:**
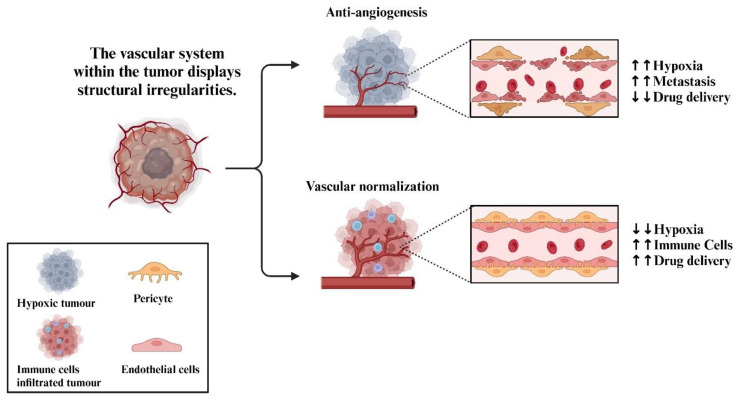
Anti-angiogenesis vs. vascular normalisation. The conventional treatment of cancer with anti-angiogenesis drugs is associated with negative outcomes. Despite limiting the amount of nutrition delivered to cancer cells, it can also increase hypoxia, resulting in increased metastasis. Drug delivery to the tumour site can also be restricted by anti-angiogenesis. Alternatively, vascular normalisation can reduce levels of hypoxia and enhance the tumour infiltration of immune cells and drug delivery to the tumour site. To normalise tumour vasculature and limit tumour growth, restoring pericyte support is essential. It has been demonstrated in PDA models that increasing pericyte coverage can reduce hypoxia and increase drug delivery [38].

In the TME of pancreatic cancer, the accumulation of fibrotic stroma attributes to a robust desmoplastic response which increases the interstitial fluid pressure [28]. The elevated interstitial pressure compresses stromal blood vessels leading to hypovascular and hypoxic microenvironments [29], which may contribute to the unsatisfactory outcomes observed in anti-VEGF treatment [17]. In murine models of pancreatic neuroendocrine carcinoma and glioblastoma, angiogenesis inhibitors targeting the VEGF pathway have been shown to partially inhibit tumour growth, while causing increased invasiveness and elevated metastasis [30]. The increased invasiveness and metastasis triggered by anti-angiogenic treatment have also been observed in other types of tumours [31,32,33,34]. Furthermore, certain angiogenesis inhibitors, particularly broad-spectrum TKIs, have the capacity to concomitantly affect MVD and microvessel integrity (MVI), which decrease pericyte coverage around the blood vessels [34]. The reduction in coverage of vascular pericytes leads to the destabilisation of vessels, resulting in increased permeability and promoting metastasis [35]. Conversely, the vascular normalisation approach induces a more physiologically balanced state within the TME, resulting in better outcomes of patients with reduced adverse effects [36]. More importantly, the vascular normalisation approach contributes to optimising the effectiveness of radiotherapy, chemotherapy, and immunotherapy, thereby holding potential to bring about more favourable clinical outcomes [37] (Figure 2).

Preclinical studies have shown that tumour vasculature normalisation decreased hypoxia and thus metastasis while increasing drug delivery to the tumour site, enhancing the anti-tumour effect of the drug [38]. Tumour vasculature normalisation has been shown to enhance the anti-tumour immune response in other cancers [39,40]. Semaphorin 3A (SEMA3A) contributes to the physiological vascular normalisation via the negative regulation of integrins [41]. In pancreatic cancer, inhibiting STAT3 along with gemcitabine effectively increased MVD and facilitated the delivery of gemcitabine to tumours without changes in the stromal collagen or hyaluronan levels [42]. In breast cancer, Salvianolic acid B (SalB) can normalise blood vessels by stimulating the interactions between tumour cells and endothelial cells. As a result, cisplatin was delivered more effectively into the tumour, and tumour metastasis was decreased [43].

Histidine-rich glycoprotein (HRG), a highly prevalent and extensively characterised protein in the plasma [44], facilitated the stimulation of anti-tumour immune responses and the normalisation of blood vessels, which in turn decreased tumour growth and metastasis and increased the efficacy of chemotherapy [45]. In a separate study, a regulator of G-Protein Signalling 5 (RGS5), a major gene responsible for aberrant vascular architecture in cancer [46,47], triggered the maturation of pericytes, leading to vasculature normalisation and subsequently, reductions in both tumour hypoxia and vessel permeability. These modifications in the vasculature of the TME enhanced the influx of immune effector cells into the tumour, resulting in a substantial extension of mouse survival [47]. Table 1 provides a summary of the research studies conducted on diverse molecular targets aimed at normalising blood vessels in cancer.

The TME of pancreatic cancer contains an inefficient and leaky vasculature associated with poor tumour perfusion, leading to hypoxia and tumour metastasis. The failure of anti-angiogenesis drugs in the treatment of pancreatic cancer has urged the approach of tumour vasculature normalisation. PAKs have played important roles in endothelial cell proliferation, migration, and permeability, as well as in angiogenesis in cancer settings [48,49]. In the subsequent section, we will present an overview of the roles of PAKs in PDA-associated vasculature and the implication in therapeutic responses.

**Table 1:** Molecules used to normalise vessels and their effects and outcomes.

Cancer	Treatment	Effect	Result	Ref.
Pancreatic	Inhibition of STAT3+ gemcitabine	↑ The number of blood vessels (CD31). ↓ Cytidine deaminase (cda) expression. ↓ Collagen alignment. ≈ Hyaluronan and collagen density.	↓ Tumour weight. ↑ Mouse survival.	[42]
Breast	Salvianolic acid B+ cisplatin	↑ αSMA/CD31 area, NG2/CD31 area (pericyte coverage), Col IV/CD31 area. ↑ VE-cadherin/CD31 area, Lectin, and O2 average. ↓ Dextran%, PIMO, and HIF-1α.	↓ Hypoxia. ↓ Metastasis. ↑ Chemotherapy and drug penetration to the tumour.	[43]
Hepatocellular	HRG via downregulation of placental growth factor (PIGF)	↑ Vessel perfusion (Lectin + CD31 + Vessels). ↓ Haemorrhage. ↑ Pericyte coated vessels.	↓ Hypoxia. ↓ Tumour cell apoptosis. TAM polarisation ↑ T cells (CD8+), dendritic cells (CD11c+), and NK cells. ↓ Necrosis.	[45]
Hepatocellular	Tanshinone IIA	↑ Tube formation. ≈ Microvessel density (MVD). ↑ Microvessel integrity (NG2 expression).	↓ Metastasis.	[50]
Breast	Sinomenine hydrochloride	↑ Lectin+CD31+vessels and αSMA positive vessels (pericyte coverage). ↓ HIF-1α.	↓ Hypoxia. ↓ Necrotic area. ↓ Growth rate and metastasis.	[51]
Pancreatic	Inhibition of Rgs5	↑ Number of vessels. ↓ Vessel diameter. ↑ αSMA and NG2 (pericyte coverage).	↓ Hypoxia. ↑ CD8 T cells. ↑ Mouse survival.	[47]

## 3. PAK in Pancreatic Cancer

PDA is dominated by mutations in the oncogene of KRas, which activates downstream signalling pathways including other GTPases such as CDC42 and Rac [52], which in turn activate PAKs [53]. There are two groups containing six members of PAKs: group 1—PAK1, PAK2, and PAK3; group 2—PAK4, PAK5, and PAK6. Alterations in the expression or activity of these PAK family members have been implicated in various physiological and pathological processes, including cell proliferation, migration and invasion, autophagy, immune cells activation, chemotherapy sensitivity, and survival [54].

The most extensively studied members among the PAK proteins are PAK1 and PAK4, which have been found to be overexpressed in many types of cancers [55,56,57]. The involvement of PAK1 in PDA tumorigenesis is characterised by its intricate and multifaceted nature. PAK1 interacts with the NFκB-p65 complex, thereby enhancing the transcriptional expression of fibronectin, causing cells to transform, and promoting the invasive epithelial–mesenchymal transition (EMT) [58]. PAK1 influences the production of VEGF, a growth factor that supports tumour invasion and metastasis [59,60].

**Figure 3:**
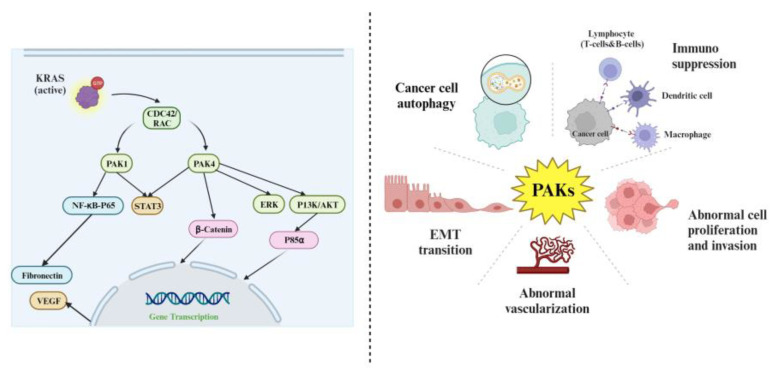
Activation and functions of PAKs in pancreatic cancer. PAK1 and PAK4 act downstream of KRas, stimulate cell proliferation, epithelial–mesenchyme transition (EMT), and migration/invasion, and contribute to abnormal vasculature and immune suppression.

PAK4 stimulates cell proliferation and survival through AKT and ERK pathways and plays a key role in regulating the proliferation of vascular smooth muscle cells (VSMCs) through the AKT pathway [61]. PAK4 activates the WNT signalling pathway via direct interaction with β-catenin, contributing to an immunosuppressive TME [62]. PAK4 also interacts with p85α, a PI3K regulatory component involved in cell migration. Inhibiting PAK4 or disrupting its connection with p85α reduces migration in pancreatic cancer cells [63,64]. PAK4 influences genes linked to stem cell traits, affecting tumour sphere formation through STAT3. Inhibiting PAK4 or STAT3 reduces stem cell-like characteristics in cancer cells, offering potential therapeutic options [65]. The details of PAKs’ function in pancreatic cancer have been reviewed elsewhere. We have provided a summary of PAK roles in pancreatic cancer broadly in Figure 3.

The unique dense stromal which exists in the TME of PDA compresses vasculatures and reduces blood vessel function, preventing the effective delivery of drugs into tumour sites and inhibiting the immune cell penetration to the tumour site. The regulation of tumour vasculature will have a significant impact on the efficacy of chemo- and immune-therapies. PAKs have recently gained attention in angiogenesis and the modulation of vasculature in cancer settings, including PDA [66,67,68,69]. Below, we will discuss the role of PAK 1 and PAK4 in tumour vasculature.

## 4. PAK and Tumour Vasculature

Rho, Rac, and Cdc42, members of the Rho family of small GTPases, are responsible for the rearrangement of actin cytoskeleton in cells and regulate integrin adhesion complexes located at the cell surface in response to various signals [70]. They also play integral roles in governing endothelial cell dynamics, (including proliferation, polarisation, intercellular adhesion, and migration), vascular permeability, and angiogenesis [71,72]. It has been shown that mice with endothelial-specific CDC42 knockout could not survive until birth owing to the lack of formation of blood vessels during the development of embryos, indicating the important role of CDC42 in the development of microvessels and vascular permeability [73]. Endothelial cells depleted of CDC42 formed a smaller tube, and the number of tubes formed and branching points were also decreased [74]. PAKs acting downstream of CDC42 have become the focus of research on the regulation and/or modulation of vasculature, particularly in cancer settings.

PAK1 has a vital role in the process of tumour angiogenesis. PAK1 amplification has been associated with advanced tumour stages, higher MVD, and notable colony stimulating factor 2 expression (CSF2) in myxofibrosarcoma. CSF2 is a cytokine that regulates the differentiation of macrophages and promotes angiogenesis, which is downregulated in PAK1 knockdown groups [75]. In breast cancer, PAK1 has been recognised as an essential element to stimulate VEGF production following the activation of human epidermal growth factor receptor signalling by Heregulin β1 (HRG). This process promotes the formation of new blood vessels [76]. The activation of PAK1 in a human cholangiocarcinoma cell impedes the degradation of hypoxia-inducible factor 1 alpha (HIF-1α) protein. This leads to the accumulation of HIF-1α, which in turn triggers the elevation of VEGF, consequently stimulating the process of tumour angiogenesis [77,78]. In a xenografted model of prostate cancer, inhibition of PAK1 decreased tumour growth, which was associated with a 70% reduction in laminin-positive blood vessels. This reduction in vascular density was associated with a tenfold decrease in matrix metalloproteinases 9 (MMP9) expression [79]. VEGF and angiogenesis are directly associated with the expression of MMPs [80].

In the TME of pancreatic cancer, there is a positive correlation between PAK1 expression and the expression of α-SMA and Desmin, suggesting the potential involvement of cancer-associated fibroblasts (CAFs), which are known contributors to angiogenesis [81]. PAK1 protein expression levels have been found to correlate positively with αSMA and Desmin in pancreatic stellate cells (PSCs) [82], which stimulate angiogenesis and facilitate tumour extravasation. These PSCs accompany circulating cancer cells, creating a vital microenvironment that facilitates primary cancer cells in establishing metastatic colonies [83]. Our preliminary data from a comprehensive global proteomic analysis showed a substantial decrease in fibronectin levels in the PAK1 knockout (KO) pancreatic cancer cells. This finding is of significant interest as fibronectin plays a pivotal role in promoting angiogenesis, a process crucial for the advancement of PDA [84].

PAK4 plays a critical role in coordinating the abnormal development of blood vessels and the exacerbation of low-oxygen (hypoxic) conditions within a tumour. In a kinome-wide screening of mesenchymal-like transcriptional activation in human glioblastoma-derived endothelial cells, PAK4 was identified as a selective regulator of genetic reprogramming in tumour vasculature, leading to aberrant vascularisation [85]. PAK4 knockout recovered the expression of adhesion proteins in tumour-derived endothelial cells, leading to normalisation of vasculature, which was associated with increased T cell infiltration and decreased tumour growth. PAK4 was found to reprogram the endothelial transcriptome and downregulate claudin-14 and VCAM-1 expression, enhancing vessel permeability and reducing T cell adhesion to the endothelium [85]. PAK4-regulated endothelial cell plasticity contributes to the modification of the vascular microenvironment and thus the efficacy of cancer therapy.

In prostate cancer, inhibition of PAK4 increased the width of blood vessels in the tumour context [86]. Furthermore, the blood vessel density stained by PECAM1 (CD31) showed a trend of elevation in the PAK4 knockdown (KD) tumour, despite not reaching statistical significance. The RNA expression of ICAM1 and VCAM1 genes also increased in the PAK4KD group [86]. Leukocyte transmigration and endothelial permeability are dependent on ICAM-1 expression [87,88,89]. Upon stimulation with cytokines, VCAM-1 is released from the endothelium and binds to integrins α4β1 and α4β7 on leukocytes. In addition to being involved in leukocyte adhesion and rolling, VCAM-1 also plays a role in leukocyte extravasation to some extent; however, its role in regulating vascular permeability has not been clarified [88,90]. In melanoma, Cercam1, Enpep, Itga3, and Lgals3 expressions were higher in tumours with PAK4 inhibition, indicating changes in angiogenesis [91].

The roles of PAKs in tumour vasculature are summarised in Table 2. Vascular changes will have a substantial impact on the effectiveness of chemotherapy and the infiltration of immune cells into the tumour. In the subsequent discussion, we will provide a summary of research findings that highlight the significance of PAKs inhibition in relation to chemotherapy responses and the activation of immune cells.

## 5. PAK and Therapeutical Responses

In pancreatic cancer, the extensive desmoplastic reaction leads to the formation of a dense stroma, causing inadequate vascularisation, inefficient drug delivery, and ineffective immune cell infiltration into the tumour sites, which eventually leads to therapeutical resistance and cancer progression [93]. PAKs have been recognised for their roles in the regulation of vasculature and therapeutical response. Here, we will discuss the impact of PAK effects on tumour vasculature in chemotherapy and immune response with a focus on pancreatic cancer.

### 5.1. PAK and Chemotherapy

PAKs are activated and play a crucial role in facilitating the resistance of cancer cells [94,95]. Modulating PAK activity holds the promise of sensitising cancer cells to chemotherapeutic agents, ultimately leading to an improved response to treatment. Shikonin, a natural inhibitor of PAK1, sensitises the pancreatic cancer cells to the chemotherapeutic drugs of gemcitabine and 5-FU [96]. A PAK1 inhibitor, FRAX597, when combined with gemcitabine, led to a synergistic inhibition of pancreatic cancer proliferation in vitro and a further reduced tumour growth in vivo [97]. The structural normalisation of blood vessels through PAK4 inhibition can enhance drug distribution and mitigate intra-tumoral hypoxia, ultimately resulting in enhanced tumour responses to molecular targeted therapy as well as radiotherapy and chemotherapy [85]. Inhibition of PAK4 not only reduces tumour growth but also enhances the efficacy of gemcitabine in mice carrying pancreatic cancer [98]. The combination of the PAK4 inhibitor, KPT-9274, and NAMPT modulators (KPT-9307) more effectively suppressed tumour growth in a xenografted mouse model [99]. The effects of PAK inhibition on the chemotherapy response of cancer cells are summarised in Table 3.

### 5.2. PAK in Tumour Immune Responses and Immunotherapy

Tumour vasculature normalisation would potentially facilitate immune cell infiltration and increase the efficacy of immunotherapy. Immune checkpoint inhibitors have been found to enhance vessel normalisation by engaging CD4+ T lymphocytes [100]. PD-L1 expression is upregulated via the activation of the PI3K/AKT and interferon-γ pathways, both of which have close interactions with PAK1 [59,101,102]. We have reported that PAK1 inhibition increased levels of intra-tumoral CD3+, CD4+, and CD8+ T cells and decreased PD-L1 expression of cancer cells, thereby stimulating anti-tumour immunity [81]. The observed diminished protein expression levels of αSMA and Desmin in the PAK1KO tumours suggest a potential impact on the deactivation of cancer-associated fibroblasts, which in turn may lead to the anticipated reduction in angiogenesis within the PAK1KO tumours [4,81].

PAK4 expression is negatively correlated with immune cell infiltration in other cancers. High PAK4 expression is associated with limited infiltration of T cells and dendritic cells in immune checkpoint inhibitor-resistant melanoma patients. However, genetic deletion of PAK4 reversed PD-1 blockade resistance by increasing CD8 T cell infiltration in mice carrying flank tumours of melanoma. A combination of anti-PD-1 treatment and a PAK4 inhibitor enhanced anti-tumour responses compared to anti-PD-1 treatment alone. The effects of PAK4 KO were mediated via inhibition of the WNT pathway [103]. Wnt/β-catenin signalling is involved in fundamental vascularisation processes, including sprouting and non-sprouting angiogenesis, vasculogenic mimicry, and the formation of mosaic vessels [104].

Inhibition of PAK4 enhanced the infiltration of CD103+ dendritic cells and CD8 T cells, and its expression correlates with CCL21 levels in melanoma biopsies. Notably, PAK4 KO cells exhibited increased expression of genes associated with blood vessel formation, including Cercam, Enpep, Itga, and Lgals. CD31, a primary blood vessel marker, was increased in tumours treated with anti-PD-1 in PAK4 KO mice [91]. In glioblastoma (GBM), inhibition of PAK4 has induced intra-tumoral CD3+ T-cell infiltration, rendering GBM more susceptible to the immunotherapy of CAR-T cells. It has been demonstrated that PAK4 inhibition transformed the disordered morphology of the tumour-associated vasculature, characterised by tortuous and disjointed vessels with spatial heterogeneity, into a well-organised structure marked by continuous vessels [85]. A similar effect has been observed in prostate cancer, where targeting PAK4 increases the infiltration of CD8 T cells and B cells into the tumour, reversing PD-1 blockade resistance. Inhibiting PAK4 also leads to increased angiogenic activities and increased expression of adhesion molecules in the TME, attracting CD8+ lymphocytes [86]. The effects of PAK1 and PAK4 on the tumour infiltration of immune cells and the associated changes in tumour vasculature have been summarised in Table 4.

## 6. Conclusions

Tumour vasculature plays a significant role in regulating the supply of nutrients, oxygen, and immune cell infiltration in the TME. Efforts in anti-angiogenesis treatments, designed to deprive cancer cells of nourishment by diminishing tumour blood vessel formation, have yielded outcomes below optimistic projection. The reduction in tumour vascularisation results in increased tumour hypoxia, leading to increased therapeutical resistance and cancer metastasis. The vasculature normalisation approach aims to reinstate proper blood flow, thereby improving the distribution of therapeutic drugs, enhancing the tumour infiltration of immune cells, and decreasing the spread of metastasis [105], which has important implications in pancreatic cancer where anti-angiogenesis treatment has limited effect.

**Figure 4:**
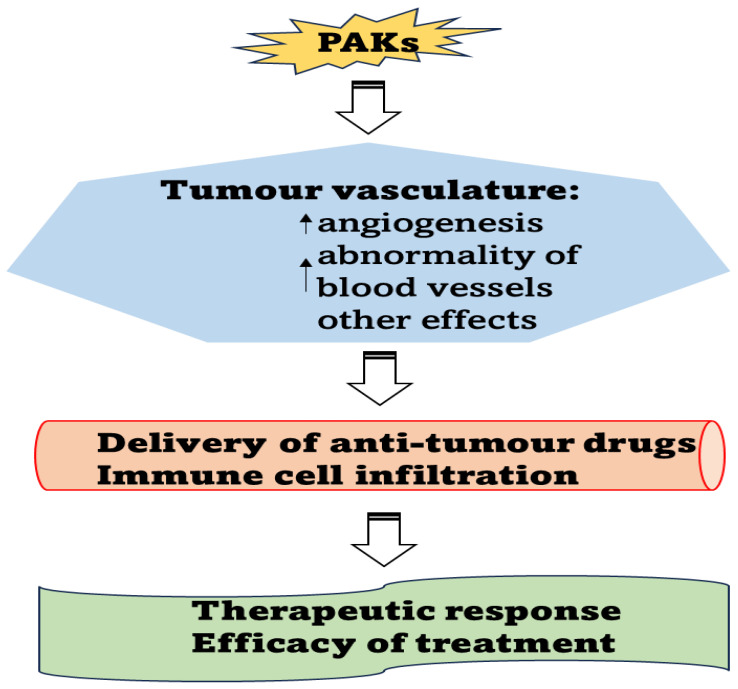
Significance of the effects of PAKs on tumour vasculature in cancer treatment.

In view of the importance of the immune system in cancer, it is crucial to understand the molecular mechanisms involved in immune cell infiltration and vasculature changes. The interaction between vasculature, tumour immune response, and PAKs in cancer biology presents a complicated and dynamic landscape. As simplified in Figure 4, the advancement in further exploring how PAKs affect tumour vasculature and change the tumour immune response will stimulate the development of novel strategies for the treatment of pancreatic cancer to improve patients’ survival.

## Figures and Tables

**Table 2:** PAKs in tumour vasculature.

Type of Cancer	Treatment	Result	Other	Ref.
Myxofibrosarcoma	↑ PAK1	↑ Tube formation and, ↑ Microvascular density (CD31 expression).	↑ CSF2 expression.	[75]
Breast	↑ PAK1	↑ Angiogenesis (CD34 expression).	↑ VEGF.	[76]
Cholangiocarcinoma	↓ PAK1	↓ Tube formation and angiogenesis.	↓ VEGF.	[77]
Melanoma	↓ PAK1	↓ Tube formation and number of blood vessels.	PAK1 expression is regulated by JMJD6, which phosphorylates RAF and MEK and positively regulates the MAPK pathway.	[92]
Prostate	↓ PAK1	↓ Laminin positive blood vessels.	↓ MMP9 (tenfold).	[79]
Pancreatic	↓ PAK1	↓ αSMA and Desmin.	Deactivation of PSCs contributing to angiogenesis.	[81,82]
Glioblastoma endothelial cells	↓ PAK4	↑ Tube formation. ≈Change of CD31 expression. ↓ Monolayer permeability. ↓ Hypoxia and vascular abnormality.	↓ Fibroblast-specific protein 1 (FSP-1), αsma, CDH2 (N-cadherin), SLUG, and ZEB1. ↑ Claudin-1, Claudin-14, ICAM1, and VCAM1 expression (twofold).	[85]
Prostate	↓ PAK4	↑ Blood vessel width and density (PECAM1).	↑ ICAM1 (CD54), VCAM1 (CD106), VEGFA RNA sequencing, and PECAM1 (CD31).	[86]
Melanoma	↓ PAK4	↑ Cercam1, Enpep, Itga3, and Lgals3.		[91]

**Table 3:** Inhibition of PAKs enhances cancer cells’ response to chemotherapy drugs.

Cancer Type	PAK Inhibitors	Target	Treatment	Mechanism	Ref.
Melanoma	PF-3758309	PAK1 PAK4	BRAFi inhibitor MEKi inhibitor	Enhance apoptosis and reduce cellular resistance to combined therapy via regulation of multiple pathways including JNK, ERK, β-catenin, and mTOR.	[95]
Pancreatic	Shikonin	PAK1	Gemcitabine 5-FU	Increase apoptosis in cancer cells.	[96]
Pancreatic	FRAX597	PAK1	Gemcitabine	Suppress HIF1α and AKT.	[97]
Pancreatic	PF-3758309	PAK1 PAK4	Gemcitabine 5-FU Abraxane	Reduce cell proliferation and enhance apoptosis. Decrease the expression of αSMA, Phalloidin, and HIF1α in vivo.	[100]
Pancreatic	PAKib	PAK4	Gemcitabine	Induce cell cycle arrest and cell death and regulate cell junction and adhesion.	[98]
Pancreatic	KPT-9274 KPT-9307	PAK4	Gemcitabine	Downregulate p-Bad-microRNA drug resistance axis and upregulate tumour-suppressive miRNAs.	[99]

**Table 4:** Inhibition of PAKs causes the infiltration of immune cells into the tumour area.

Type of Cancer	Treatment	Techniques	Type of Immune Cell Activation	Possible Effect on Tumour Vasculature	Ref.
Pancreatic	↓ PAK1	Western blot and IHC	T cells (CD3+, CD4+, and CD8+)	↓ αSMA and Desmin, which indicates cancer associated fibroblast deactivation, in turn anticipating the reduced angiogenesis.	[81]
Melanoma	↓ PAK4	Mass cytometry and IHC	T cells (CD8+) and dendritic cells	↑ Activation of WNT signalling pathway, anticipating the increasing angiogenesis.	[103]
Melanoma	↓ PAK4	Transcriptomic, IHC, and flow cytometry	T cells (CD8+) and dendritic cells (CD103+)	↑ Alterations in genes associated with blood vessel formation, specifically, Cercam, Enpep, Itga, and Lgals.	[91]
Glioblastoma	↓ PAK4	IHC and bioluminescence imaging	T cells (CD3+)	↑ Vascular normality with reduced hypoxia.	[85]
Prostate	↓ PAK4	Flow cytometry and IHC	T cells (CD8+) and B cells	↑ ICAM1 and VCAM1. ↑ Inflammatory signals (CCR7, CCL19 and CCL21, CXCL13, and CXCR5).	[86]

## Abbreviations

PAKs	P21-activated kinases
PDA	Pancreatic ductal adenocarcinoma
TME	Tumour microenvironment
CSCs	Cancer stem cells
5-FU	5-fluorouracil
TKIs	Tyrosine kinase inhibitors
PFS	Progression-free survival
OS	Overall survival
MVD	Microvessel density
GTP	Guanosine triphosphate
MVI	Microvessel integrity
SEMA3A	Semaphorin 3A
SalB	Salvianolic acid B
HRG	Histidine-rich glycoprotein
RGS5	G-Protein Signalling 5
Cda	Cytidine deaminase
EMT	Epithelial–mesenchymal transition
VSMCs	Vascular smooth muscle cells
CSF2	Colony stimulating factor 2
HRG	Heregulin β1
HIF-1α	Hypoxia-inducible factor 1 alpha
JMJD6	Jumonji domain-containing protein 6
MMP9	Matrix metalloproteinases 9
CAFs	Cancer-associated fibroblasts
PSCs	Pancreatic stellate cells
KO	Knockout
KD	Knockdown
GBM ECs	Glioblastoma multiforme endothelial cells
IHC	Immunohistochemical
